# The effects of opioids on HIV reactivation in latently-infected T-lymphoblasts

**DOI:** 10.1186/1742-6405-11-17

**Published:** 2014-07-02

**Authors:** Johannes Prottengeier, Eleni Koutsilieri, Carsten Scheller

**Affiliations:** 1 Department of Anesthesiology, Erlangen University Hospital, Krankenhausstrasse 12, 91054 Erlangen, Germany; 2 Institute of Virology and Immunobiology, University of Würzburg, Würzburg, Germany

**Keywords:** HIV, Reactivation, Opioids, Heroine, Morphine, Naloxone, ACH-2

## Abstract

**Background:**

Opioids may have effects on susceptibility to HIV-infection, viral replication and disease progression. Injecting drug users (IDU), as well as anyone receiving opioids for anesthesia and analgesia may suffer the clinical consequences of such interactions. There is conflicting data between *in vitro* experiments showing an enhancing effect of opioids on HIV replication and clinical data, mostly showing no such effect. For clarification we studied the effects of the opioids heroin and morphine on HIV replication in cultured CD4-positive T cells at several concentrations and we related the observed effects with the relevant reached plasma concentrations found in IDUs.

**Methods:**

Latently-infected ACH-2 T lymphoblasts were incubated with different concentrations of morphine and heroine. Reactivation of HIV was assessed by intracellular staining of viral Gag p24 protein and subsequent flow cytometric quantification of p24-positive cells. The influence of the opioid antagonist naloxone and the antioxidants N-acetyl-cysteine (NAC) and glutathione (GSH) on HIV reactivation was determined. Cell viability was investigated by 7-AAD staining and flow cytometric quantification.

**Results:**

Morphine and heroine triggered reactivation of HIV replication in ACH-2 cells in a dose-dependent manner at concentrations above 1 mM (EC_50_ morphine 2.82 mM; EC_50_ morphine 1.96 mM). Naloxone did not interfere with heroine-mediated HIV reactivation, even at high concentrations (1 mM). Opioids also triggered necrotic cell death at similar concentrations at which HIV reactivation was observed. Both opioid-mediated reactivation of HIV and opioid-triggered cell death could be inhibited by the antioxidants GSH and NAC.

**Conclusions:**

Opioids reactivate HIV *in vitro* but at concentrations that are far above the plasma levels of analgesic regimes or drug concentrations found in IDUs. HIV reactivation was mediated by effects unrelated to opioid-receptor activation and was tightly linked to the cytotoxic activity of the substances at millimolar concentrations, suggesting that opioid-mediated reactivation of HIV was due to accompanying effects of cellular necrosis such as activation of reactive oxygen species and NF-κB.

## Introduction

Many of the HIV-infected individuals who inject intravenous drugs consume morphine, heroin or related substances. There is conflicting data on the impact of opioids on HIV disease progression. Whereas in vitro studies suggest a potential link between opioid exposure and elevated virus replication or decreased immune function [1-5], most of epidemiological and clinical studies do not find a correlation between opioid intake and progression of HIV infection [6-12]. In order to reconcile these conflicting data, we performed *in vitro* experiments studying the effects of opioids (heroin and morphine) on HIV replication in the chronically-infected CD4-positive T cell line ACH-2 [13]. CD4-positive T cells represent the cellular reservoir in which HIV is harbored predominantly in the body and they contribute to most of the viral replication detected in the blood plasma [14-16].

## Results

### Morphine and heroine reactivate HIV in ACH-2 cells

**Figure 1:**
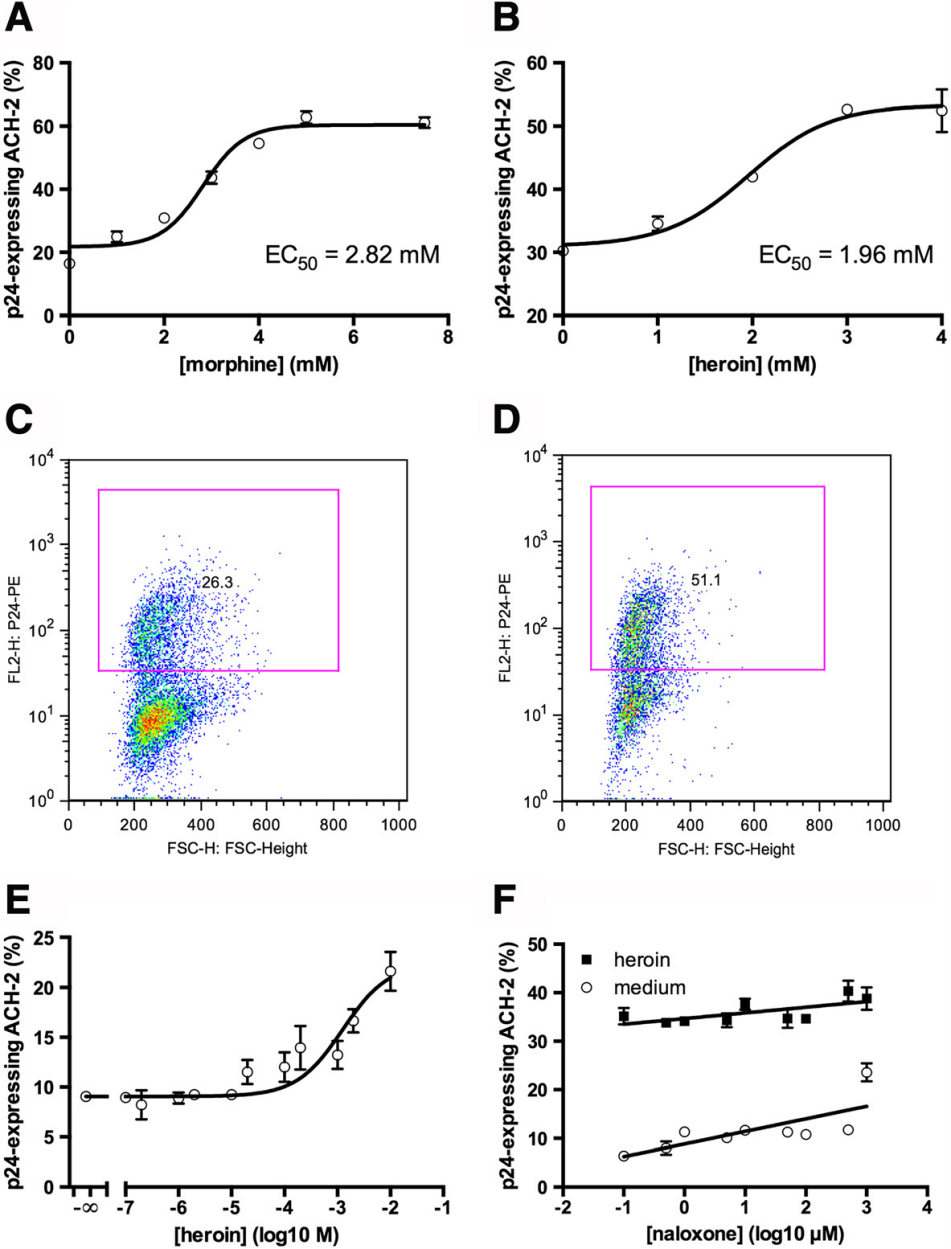
**Opioids activate HIV replication in vitro.** Latently-HIV-infected ACH-2 T lymphoblasts were cultured for 24 hours in the presence of different concentrations of morphine **(A)** or heroin **(B)**. HIV replication was quantified by intracellular staining of HIV p24-antigen and flow cytometry. **C**, **D**: representative dot-plot analyses of ACH-2 cells left untreated **(C)** or treated with 4 mM heroin **(D)**. The x-axis displays the forward scatter, the y-axis displays p24-expression. Pink rectangles represent the gates for p24-positive cells and the small numbers within indicate the proportion (%) of p24-positive cells from the total number of events. **E**: Heroin has no effects on HIV reactivation at concentrations found in plasma of IDUs (1-10 μM). **F**: Latently-HIV-infected ACH-2 T lymphoblasts were cultured for 24 hours in the presence of 5 mM heroin, together with different concentrations of naloxone (0 μM and 0.1μM – 1 mM). HIV replication was quantified by intracellular staining of HIV p24-antigen and flow cytometry. Data as mean ± S.E.M. from duplicates and linear regression. The slopes of both regressions are positive and significantly differ from = zero (p = 0.0008 for medium and p = 0.0152 for heroin).

In order to investigate the effects of the opioids heroine and morphine on reactivation of proviral HIV in latently-infected T cells, we incubated ACH-2 cells with different concentrations of morphine-sulfate and heroine. After incubation for 24 h we measured intracellular HIV-p24 protein expression by flow cytometry. We found a dose-dependent reactivation of HIV for morphine (Figure 1A) and heroine (Figure 1B). The EC_50_ concentrations for the two substances were 2.82 mM and 1.96 mM, respectively (Figure 1). Figures 1C and D display representative dot-plots analyzing p24-expression (y-axis) of untreated (Figure 1C) or 4 mM heroin-treated (D) cells. The background expression visible in Figure 1C is typical for ACH-2 cells and originates from intrinsic stimulation of the cells. We also tested the effects of heroin on HIV replication at very low concentrations, similar to the plasma concentrations found in IDUs and found no activating effects (Figure 1D).

### Naloxone does not inhibit heroine-mediated reactivation of HIV

Opioid receptor signaling has been described on lymphocytes [1] and opioid receptors that bind the opioid antagonist naloxone have been identified on T cells (reviewed in [17]). We therefore investigated whether the observed HIV-activating effect of opioids was mediated by opioid-specific receptor stimulation. For that we triggered HIV reactivation with heroine in the absence or presence of different concentrations of naloxone. As depicted in Figure 1F, naloxone did not interfere with heroine-mediated HIV reactivation, indicating that opioid receptor-independent mechanisms account for the observed effects.

### NAC and GSH inhibit opioid-mediated HIV reactivation

**Figure 2:**
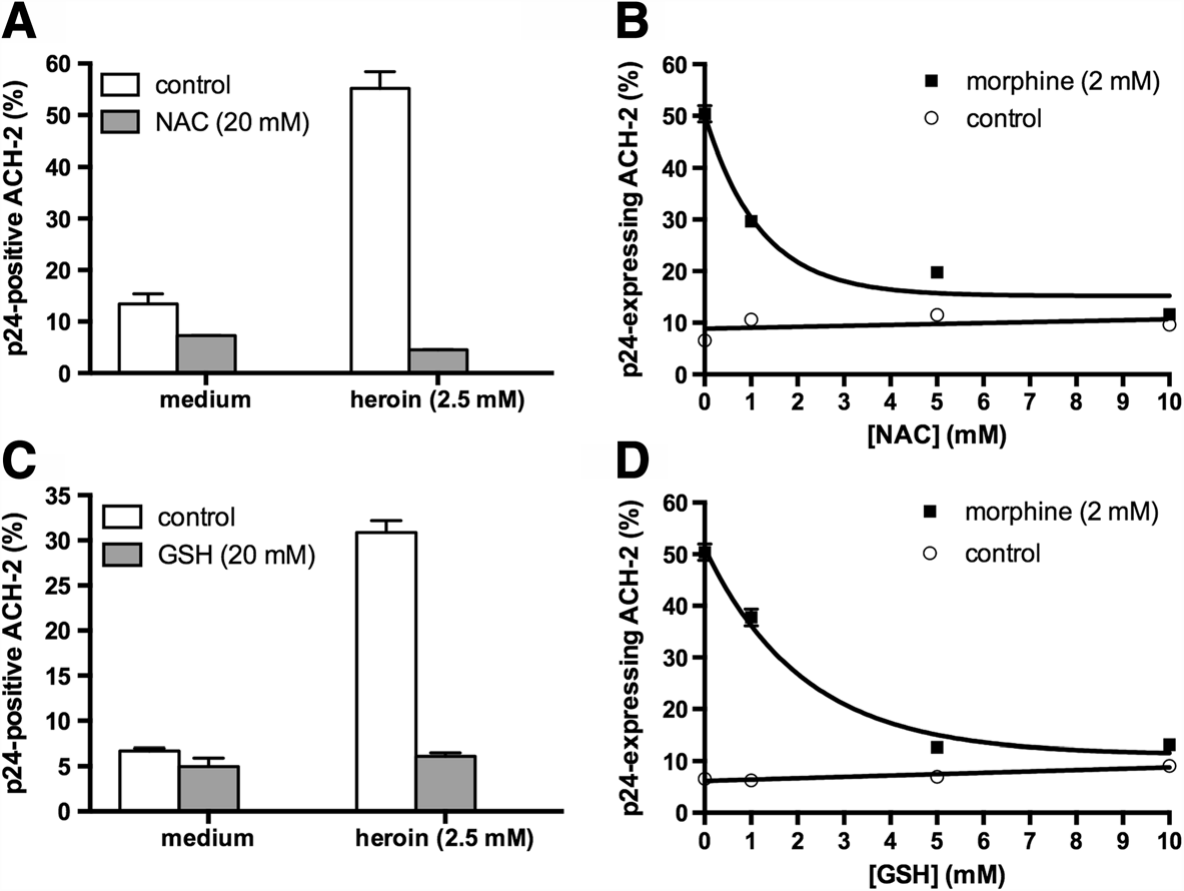
**Antioxidants inhibit opioid-mediated reactivation of HIV.** Latently-HIV-infected ACH-2 T lymphoblasts were cultured for 24 hours with of heroin **(A, C)** or morphine **(B, D)** in the presence or absence of the antioxidants N-acetylcystein (NAC) **(A, B)** or glutathione **(C, D)**. HIV replication was quantified by intracellular staining of HIV p24-antigen and flow cytometry.

**Figure 3:**
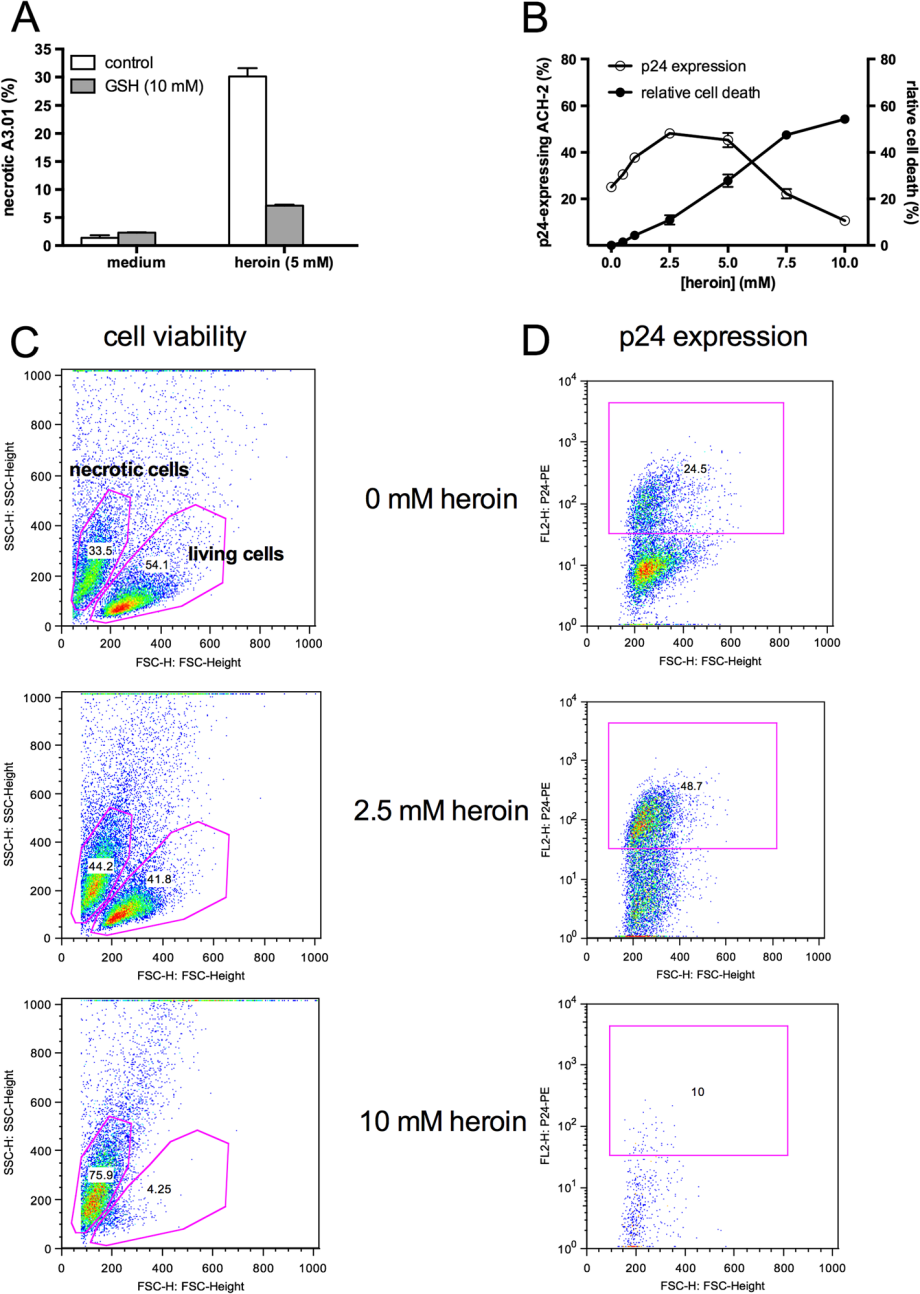
**Opioid-mediated reactivation of HIV is tightly linked to cellular necrosis.** Latently-HIV-infected ACH-2 T lymphoblasts were cultured for 24 hours with of heroin in the presence or absence of glutathione. HIV replication was quantified by intracellular staining of HIV p24-antigen and flow cytometry **(A)**. Cell viability was assessed by FSC/SSC-analysis in flow cytometry **(B)**. Representative dot plots of cell viability analysis **(C)** and p24-expression **(D)** at different heroin concentrations. Pink rectangles on the right represent the gates for p24-positive cells and the small numbers within indicate the proportion (%) of p24-positive cells from the total number of events. Pink regions on the left represent the gates for living and necrotic cells and the small numbers within indicate the proportion (%) of events within the region from the total number of events.

Heroin- and morphine-mediated activation of HIV replication was completely prevented by the antioxidants N-acetylcystein (NAC) or reduced glutathione (GSH) (Figure 2), indicating that oxidative stress might be involved in opioid-mediated reactivation of HIV. The relatively high opioid concentrations needed to stimulate HIV replication were also associated with induction of necrotic cell death. A3.01 T cells, the uninfected parental cell line of ACH-2 cells, showed a significant amount of necrosis induction following treatment with 2.5 mM heroin as assessed by staining with the cell dye 7-AAD (Figure 3A). This necrosis induction could again be completely prevented by the antioxidant N-acetylcystein (Figure 3A). We used for this experiment the uninfected parental cell line because the 7-AAD dye requires nonfixated cells, which in case of the HIV-infected ACH-2 cells we could not subject to our flow cytometer for safety reasons. In order to demonstrate that ACH-2 cells react in a similar way to opioids as A3.01 cells, we analyzed cell death in ACH-2 cells by a characteristic shift of the cell population in the forward-scatter/sideward-scatter dot plot analysis, which correlates with 7-AAD-staining. This shift is depicted in Figure 3C from representative analyses for different heroin concentrations. The corresponding p24-expression profiles are depicted in Figure 3D. As shown in Figure 3B, activation of HIV in ACH-2 cell completely paralleled with opioid-triggered necrosis. We reported earlier that necrotic signaling triggers NF-κB and HIV-reactivation in ACH-2 T lymphoblasts [18]. These data indicate that the observed opioid-mediated activation of HIV replication was secondary to the opioid-triggered necrosis observed at high concentrations.

## Discussion

Opioid-mediated reactivation of latent HIV infection in our model was observed at concentrations in the lower millimolar range. Since opioids are common in the HIV-risk group of injecting drug users (IDUs), this activity might influence the pathogenesis of HIV infection in this group of patients. However, plasma concentrations of heroine in IDUs are in the range of 1–10 μM [19] and even maximum plasma concentrations are only in the range of 1500–3900 ng/ml (reviewed in [20]), which corresponds to 4.1-10.7 μM (calculated with a molar weight of 369.41 g/mol). Secondly plasma-concentrations of medically administered morphine and its modern derivates for means of analgesia remain well below those ranges of reactivating effects [21-23]. These *in vivo* concentrations are 2–3 orders of magnitude lower than the *in vitro* concentration at which we observed a stimulating effect. It therefore seems very unlikely that the here-described opioid-mediated reactivation of HIV may have any influence on HIV replication *in vivo*.

Opioid-triggered reactivation of HIV replication in latently-infected cells has been described in the monocytic cell line U1 [3]. HIV reactivation after treatment with morphine was observed in the picomolar (10^-12^) range but not at higher concentrations. Especially at micromolar concentrations – the concentration range of plasma levels found in drug abusers - no effects were observed [3]. Similar effects were reported from the same group when studying productive infection in cultured peripheral blood lymphocytes [4]. In studies with experimental SHIV-infection in the rhesus macaque model, elevated viral load was reported in morphine-treated animals (n = 6) versus untreated animals (n = 3) [2]. Recently, a decrease in anti-HIV microRNA expression was reported in monocytes following treatment with morphine and heroin [5], suggesting a potential explanation for the increased viral replication found in *in vitro* studies.

In contrast, the majority of epidemiological studies does not show negative effects of drug abuse on HIV progression [8-12], but many confounding variables such as the pattern of drug use may make it difficult to clearly interpret this data. To address this, a prospective clinical study with a total of 1148 participants was performed, in which the effects of hard drug use (opioids or cocaine) on HIV disease parameters was studied [6]. Drug use had no effect on HIV viral load or CD4 cell counts (n = 613 versus n = 535). In line with this, no effects of drug use on viral load have been observed in a seroconverter study with 60 injection drug users in a total of 149 participants [7].

The literature therefore seems to be divided into experimental and *in vitro* studies reporting enhancement of HIV infection following treatment with opioids and *in vivo* studies not finding such a correlation. We suggest that this difference, at least in parts, relates to the concentration range at which effects were observed: All *in vitro* studies reporting an HIV-activating effect of opioids in cell culture found these effects at very low concentrations and no activity was observed at relevant plasma concentrations [3-5]. Moreover, all so-far reported *in vitro* data were generated in monocytes [3,5] (or in PBMC containing monocytes [4]), whereas more than 99% of HIV particles detected in the human plasma originate from T-lymphocytes [15,24,25]. Hence, effects found in monocytes *in vitro* may not be representative for the lymphocyte-driven plasma viral load *in vivo*. Our *in vitro-*results indicate that there is an HIV-stimulating effect of opioids also in T cells, but only at concentrations that are far above relevant *in vivo*-concentrations. The molecular mechanism of HIV latency in the ACH-2 cell line used in our study is caused by a defect in the Tat/TAR axis [26] and the same mechanism of latency is also frequently found in latently-infected primary T cells isolated from HIV patients [27].

Taken together, the literature seems to be divided into *in vitro* studies reporting enhancement of HIV infection following treatment with opioids and *in vivo* studies not finding such a correlation. Our *in-vitro* results indicate that there is an HIV-stimulating effect of opioids in latently-infected T cells, but only at concentrations that are far beyond relevant *in vivo*-concentrations and our findings may therefore contribute to reconcile the apparently conflicting data of *in vitro* and *in vivo* research. Our data provides no evidence that the medical use of opioids for indications of anesthesia and analgesia in HIV-infected patients may have any side effects on HIV replication.

## Conclusion

Opioids reactivate HIV *in vitro* but at concentrations that are far above the plasma levels of analgesic regimes or drug concentrations found in IDUs. HIV reactivation is mediated by effects unrelated to opioid-receptor activation and seems tightly linked to the cytotoxic activity of these substances at a millimolar level, suggesting that opioid-mediated reactivation of HIV is due to accompanying effects of cellular necrosis such as activation of reactive oxygen species and NF-κB.

## Materials and methods

### Cell culture

A3.01 (human CD4 + -T-lymphoblasts, NIAID) and ACH-2 (latently HIV-infected A3.01, Folks 1989, NIAID) were cultured in standard tissue flasks using RPMI-medium (Invitrogen, Karlsruhe, Germany) supplemented with 10% FCS (Linaris, Bettingen, Germany) at 37°C and 5% CO2 atmosphere. Peripheral blood mononuclear cells (PBMC) were isolated by Ficoll/Paque density-separation centrifugation from human whole blood donated by healthy volunteers. For the different experiments 10^5^ cells per well were cultured in flat-bottom 96 well plates in a total volume of 200 μl for 24 h in the absence or presence of the indicated substances.

### Opioids, antagonists and other reagents

Morphine and Heroine were obtained from the University of Wurzburg’s Department of Forensic Medicine. The Use and Discard of those substances were documented in accordance with the German Narcotics Act. Naloxone, Glutathion and N-Acetylcysteine were purchased through Sigma, St.Lewis, MO.

### Flow cytometry

Reactivation of HIV in ACH-2 cells was quantified by the expression of viral p24-antigen detected by flow cytometry (Becton-Dickinson, Heidelberg, Germany). Cells were fixated for 20 min with 4% formalin in PBS and later permeabilized with 0.5% saponin in PBS/5%BSA. The following antibodies were used: 183-H12-5C-anti-HIV-p24-mouse-antibody and goat-anti-mouse antibodies conjugated with FITC or PE (BD Biosciences). Cell death in uninfected A3.01 cells was quantified by 7-AAD staining according to the instructions of the manufacturer (BD Biosciences). Besides staining with 7-AAD, necrotic cells also displayed a characteristic shift in a forward-scatter (FSC)/sideward scatter (SSC) dot plot. This shift was used to quantify necrotic cells also for HIV-infected ACH-2 cells, for which a 7-AAD staining was not possible for safety reasons (7-AAD staining requires nonfixated cells which in case of ACH-2 cells would contaminate the flow cytometer with HIV).

## Competing interests

All of the authors declare that they have no competing interests.

## Authors’ contributions

CS conceived of the study and helped to draft the manuscript. JP conducted the study, performed the statistical analysis and is the main author of the manuscript. EK participated in the study’s design and helped to draft the manuscript. All authors read and approved the final manuscript.
